# Glycosylation changes in the globular head of H3N2 influenza hemagglutinin modulate receptor binding without affecting virus virulence

**DOI:** 10.1038/srep36216

**Published:** 2016-10-31

**Authors:** Irina V. Alymova, Ian A. York, Gillian M. Air, John F. Cipollo, Shelly Gulati, Tatiana Baranovich, Amrita Kumar, Hui Zeng, Shane Gansebom, Jonathan A. McCullers

**Affiliations:** 1 Influenza Division, National Center for Immunization & Respiratory Diseases, Centers for Disease Control & Prevention, Atlanta, GA, USA; 2 Department of Biochemistry & Molecular Biology, University of Oklahoma Health Sciences Center, Oklahoma City, OK, USA; 3 Center for Biologics Evaluation and Research, Food and Drug Administration, Silver Spring, MD, USA; 4 Department of Infectious Diseases, St. Jude Children’s Research Hospital, Memphis, TN, USA; 5 Battelle Memorial Institute, Atlanta, GA, USA; 6 Department of Pediatrics, University of Tennessee Health Sciences Center, Memphis, TN, USA

## Abstract

Since the emergence of human H3N2 influenza A viruses in the pandemic of 1968, these viruses have become established as strains of moderate severity. A decline in virulence has been accompanied by glycan accumulation on the hemagglutinin globular head, and hemagglutinin receptor binding has changed from recognition of a broad spectrum of glycan receptors to a narrower spectrum. The relationship between increased glycosylation, binding changes, and reduction in H3N2 virulence is not clear. We evaluated the effect of hemagglutinin glycosylation on receptor binding and virulence of engineered H3N2 viruses. We demonstrate that low-binding virus is as virulent as higher binding counterparts, suggesting that H3N2 infection does not require either recognition of a wide variety of, or high avidity binding to, receptors. Among the few glycans recognized with low-binding virus, there were two structures that were bound by the vast majority of H3N2 viruses isolated between 1968 and 2012. We suggest that these two structures support physiologically relevant binding of H3N2 hemagglutinin and that this physiologically relevant binding has not changed since the 1968 pandemic. Therefore binding changes did not contribute to reduced severity of seasonal H3N2 viruses. This work will help direct the search for factors enhancing influenza virulence.

Influenza A viruses (IAVs) of the H3N2 subtype entered the human population in 1968 when a reassortant virus (typified by A/Hong Kong/1/68 [HK68]), containing avian-derived hemagglutinin (HA) and polymerase basic protein 1 (PB1) gene segments and along with 6 segments from the previously circulating H2N2 viruses^1^, caused a global pandemic associated with more than one million deaths worldwide^2^. Since then, the morbidity and mortality associated with H3N2 virus infection have gradually diminished^3^,^4^. Sequence changes in the HA are believed to be among the key contributing factors.

Over that time, the HA molecule of H3N2 IAV has evolved significantly including incremental increases in the number of *N-*linked glycosylation sites on the globular head^5^. These sites support attachment of sugar molecules to the side chain amide nitrogen of Asn in the context of the conserved *N*-glycosylation sequon Asn-X-Ser/Thr where X may represent any amino acid except Pro. The HA stem region of human H3N2 viruses has had five *N-*linked glycosylation sites from the time the virus entered into the human population to present day^6^,^7^ (Fig. 1). In contrast, the globular head of HK68 HA had only two *N-*linked glycosylation sites at residues 81 and 165 (numbering is based on the mature HA molecule^6^,^7^). Human H3N2 viruses have subsequently progressively gained up to seven additional glycosylation sites on the HA globular head (Fig. 1). While some human isolates have also lost some globular head glycans over this time^5^, the general trend toward increasing glycosylation is clear. There has been no *O-*linked glycosylation (e.g. attachment of a sugar molecule to the hydroxyl group of Ser or Thr on the polypeptide chain) reported for IAV^8^. The stepwise increase in the number of *N-*linked glycosylation sites on the H3 globular head has been shown to reduce virulence of H3N2 strains^9^,^10^,^11^. We have previously demonstrated this in mice by using engineered viruses containing an extra two or four glycosylation sites on the H3N2 IAV HA. Consistent with observations in humans, a progressive reduction of morbidity, mortality, and viral lung titers in mice was observed as HA glycosylation increased. Virus neutralization by lung-resident surfactant protein D (SP-D)^11^, the respiratory tract collectin, and attenuation of H3N2 IAV infection through ER stress pathways^12^, has at least in part contributed to the observed effects. However, it is likely that this increasing glycosylation can diminish virulence by other mechanisms as well.

Influenza HA initiates infection by binding to cell-surface glycan receptors with terminal *N-*acetylneuraminic acid (Neu5Ac or sialic acid [SA]) linked to galactose via α2,3- or α2,6- linkages, which are abundant on either the avian gastrointestinal or human upper respiratory tracts (RT), respectively. Recent glycan microarray analysis of the human H3N2 IAVs binding indicated a change in HA receptor binding affinity preferences from recognition of a broad range of SA-containing receptors by earlier viruses, to recognition of mainly long linear glycan structures by more contemporary isolates^13^,^14^. Consistent with this, recent H3N2 IAVs have a reduced capacity to agglutinate a range of red blood cell types^15^,^16^,^17^,^18^ and to grow in cell cultures or embryonated chicken eggs^19^,^20^. Data from several studies suggested a link between declining morbidity and mortality associated with H3N2 infection in humans and reduced binding to SA-containing receptors^18^,^21^,^22^.

Receptor binding properties of HA are determined not only by amino acid residues forming the receptor binding site (RBS), but also by glycan interactions with HA residues near the binding pocket^23^,^24^, with the precise structure and composition of the glycans being important factors^25^. As indicated by two studies using hemadsorption^10^ and sialyl glycoconjugate binding^22^ assays, the progressive addition of glycans to specific sites (mimicking those of natural human isolates) decreased H3N2 IAV binding to its receptors, although the precise SA receptors affected by additional glycosylation were not studied. In one study, the glycosylation-related decrease in HA binding was shown to reduce the lysis of infected cells by natural killer cells, potentially providing replicative advantages (e.g. enhanced virulence) for such H3N2 viruses^22^. The other study linked glycosylation-related reduction in HA receptor binding with lower replication of H3N2 virus in mice^26^. However, in contrast to either conclusion, as well as to the suggestion of a link between reduced binding to SA-containing receptors and the declining morbidity or mortality associated with H3N2 infection in humans^18^,^21^,^22^, binding data from a collection of human H3N2 IAVs that circulated from 1968 through 2012 did not indicate a correlation between the strength of receptor binding and virulence and ability to transmit in human population^13^.

Here, we examine the receptor binding, growth in epithelial cell cultures, and virulence in mice of engineered H3N2 IAVs^11^ to understand the contribution of HA glycosylation to receptor binding of contemporary strains along with consequences for virulence. We show that modulation in receptor binding efficiency has no effect on virulence, supporting observations from others on the lack of connection between H3N2 IAV binding and virulence in humans^13^,^14^. We propose that this disconnection is in part due to universal recognition of two specific sialylated structures.

## Results

### Glycosylation modulates binding of rgHK68 viruses to sialylated glycans

The binding of H3N2 variants with different levels of glycosylation was analyzed using the Consortium for Functional Glycomics (CFG) v5.1 glycan array. Three viruses, constructed by reverse genetics (rg), were used, containing unmodified HA from wild-type HK68 (rgHK68), or HK68 HA with two (residues 63 and 126: rgHK68 + 2) or four (residues 63, 126, 133, and 246: rgHK68 + 4) additional glycosylation sites^11^. In total, the three rgHK68 viruses bound to 61 sialylated glycans on the array, with 25 being of α2,3-, 34 of α2,6-, and 2 of both SA linkages (Table 1 and Fig. 2). These glycans included full glycan structures as well as substructures. Eleven glycans were recognized by all three rgHK68 viruses. Eighteen glycans were recognized by rgHK68 and rgHK68 + 4 only, and twenty-three and nine glycans were recognized only by rgHK68 or rgHK68 + 4, respectively. The most commonly recognized structural motifs were Neu5Acα2–3(6)Galβ1–4GlcNAcβ1-, and the sialylated LacNAc repeat Neu5Acα2–6Galβ1–4GlcNAcβ1–3Galβ1–4GlcNAcβ1-.

As expected, rgHK68 showed higher binding than did its glycosylation variants, based on both the number of glycans bound and the total level of fluorescent intensity (Fig. 2A,B). It recognized a total of 52 *N-* and *O-*linked sialylated glycans on the array (Table 1 and Fig. 2A), consisting of 17 with α2,3-, 33 with α2,6-, and 2 with both SA linkages. The glycans containing terminal α2,6-linked SA were among the strongest binders on the array, with the sum of all the binding intensities for this group being 27,547 ± 3,871 relative fluorescent units (RFU; Fig. 2B). Structurally, the rgHK68 recognized multiantennary glycans (such as glycans 57, 464, 466, 481, 482, and 606; the glycan numbering corresponds to those of the CFG v5.1 glycan array) with LacNAc substitutions or LacNAc repeats (short poly LacNAc; glycans 606 and 608) as major components at the highest affinity. In terms of antennary substructures, the Neu5Acα2–6Galβ1–4GlcNAcβ1–2Mana1,3- arm of the *N-*linked glycan (glycans 319, 346, and 348) appears to be a stronger requirement for high affinity binding than the equivalent Neu5Acα2–6Galβ1–4GlcNAcβ1–2Mana1,6- arm (glycans 308, 345, and 347). The glycans containing terminal α2,3-linked SA (including those with a LacNAc motif such as glycans 237, 266, 325, 462, 483, and 600) were also bound by rgHK68, however, at lower overall affinity (8,323 ± 1,046 RFU; Fig. 2B) than those with α2,6-linkage. Of the glycans with a mixture of Neu5Acα2,6- and Neu5Acα2,3-substitution (glycans 318 and 326) only glycan 326 with a Neu5Acα2–6Galβ1–4GlcNAcβ1–2Mana1,3-arm bound strongly, consistent with the notion that this arm is a robust facilitator of receptor binding. Consistent with findings from others^13^,^14^, key structural motifs required for efficient rgHK68 binding were Neu5Acα2,6-LacNAc and poly LacNAc. Multiple antennae generally increased binding signal. The Neu5Acα2,3-capped glycans also were seen to bind strongly albeit less intensely than their Neu5Acα2,6-capped counterparts.

Adding glycans to residues 63 and 126 of the HK68 HA (rgHK68 + 2) markedly reduced virus binding to glycans on the CFG array. The virus containing the rgHK68 + 2 HA showed recognition of only four α2,3-linked SA and seven α2,6-linked SA (Table 1 and Fig. 2A), and had the lowest total fluorescence intensities of the three viruses (1,049 ± 94 RFU for α2,3- and 2,825 ± 301 RFU for α2,6-linked SAs; Fig. 2B). Among the glycans that bound both rgHK68 and rgHK68 + 2, signal was diminished ~20 to 80% in the rgHK68 + 2 virus analysis. The strongest intensity was produced by biantennary *N-*linked glycans 57, 325, 481, and 483. A striking quality of the rgHK68 + 2 virus binding pattern was a complete lack of significant interaction with compounds with a higher number of antennae, the opposite of the rgHK68 virus.

Unexpectedly, adding two further glycans to positions 133 and 246 of rgHK68 + 2 HA, to create rgHK68 + 4, restored much of the binding. The rgHK68 + 4 bound to 21 α2,6-, 15 α2,3-and two dual-linked SAs with total binding intensities of 25,956 ± 3,009 RFU, 6,610 ± 649 RFU, and 1,724 ± 160 RFU, respectively (Table 1 and Fig. 2A,B). Interestingly, adding glycans to residues 133 and 246 on the H3 globular head not only restored rgHK68 + 4 binding to some of SAs bound by rgHK68, but also allowed greater signal intensity for some glycans than detected for rgHK68 (Table 1). Among the most striking changes were increases in signal from bi- and triantennary glycans containing Neu5Acα2,3-substitutions and those with Neu5Acα2–6Galβ1–4GlcNAcβ1–2Manβ1,6 arm of the sialyl *N-*glycans. As seen for the rgHK68 virus, the rgHK68 + 4 showed strong binding signals to glycans containing both LacNAc and poly LacNAc units. Unlike rgHK68, there was no preference for Neu5Acα2–6Galβ1–4GlcNAcβ1–2Manb1,3- over Neu5Acα2–6Galβ1–4GlcNAcβ1–2Manb1,6- (compare binding to glycans 319, 346 and 348 with that to glycans 308, 345 and 347). A significant difference in binding between rgHK68 and rgHK68 + 4 was observed for glycans possessing dual SA linkages (e.g. glycans 318 and 326). In the case of rgHK68 the preference is for glycan 326 where the Neu5Acα2,6 is present on the Mana1,3 arm and the Neu5Acα2,3 is present on the Man1,6 arm. In the case of rgHK+4, the preference is for glycan 318 where the Neu5Acα2–6 is present on the Mana1,6 arm and Neu5Acα2,3 is present on the Man1,3 arm. More striking, binding of rgHK68 + 4 to Neu5Acα2,6 capped chitobiose and chitotriose (glycans 366 and 367) was nearly five times higher than that of rgHK68.

Similarly to rgHK68, both glycosylation variants (rgHK68 + 2 and rgHK68 + 4) preferentially bound to Neu5Acα2,6-linked glycans, but also retained substantial recognition of Neu5Acα2,3-linked glycans, with the proportion of α2,3- and α2,6-linked SAs being similar for all viruses (31% to 39% for α2–3-linkage, and of 55% to 63% for α2,6-linkage; Table 1 and Fig. 2B). Titrating the concentration of virus confirmed the binding patterns observed with rgHK68 viruses at standard experimental conditions (Fig. 2C).

These results demonstrate that rgHK68 viruses were able to recognize a wide range of sialylated glycans with both α2,6- and α2,3-linkages on the CFG v5.1 array (Table 1). Next, we evaluated rgHK68 viruses’ bindings to the subset of glycans on the CFG array that are biologically relevant for influenza pathogenesis in the human RT^27^. Importantly, most of the glycans on the array that are significantly bound by rgHK68 viruses were structurally related to those found in human RT. In general, the trends seen with the complete CFG v5.1 array were also seen when the analysis was limited to those glycans on the array that are present in the human RT (Table 1). Binding to the SAs within this subset was comparable for the rgHK68 and rgHK68 + 4 viruses, while binding of rgHK68 + 2 was less than 50% or 20% (based on the number of glycans bound and the level of total binding intensity, respectively) of that determined for rgHK68 or rgHK68 + 4. Virus rgHK68 + 4 uniquely recognized 3 *N-*linked glycan structures of the human RT (301, 460, and 461) that were not recognized by either rgHK68 or rgHK68 + 2. Similar to rgHK68, both glycosylation variants (rgHK68 + 2 and rgHK68 + 4) preferentially bound to α2,6-linked SAs, but also retained substantial recognition of α2,3-linked SA.

These results demonstrate that increasing *N-*linked glycosylation of HK68 HA can significantly alter (i.e. reduce or increase) virus binding to SA-containing receptors, including receptors that are present in the human RT.

### Red blood cell type modulates elution pattern of rgHK68 viruses

To evaluate the functional significance of the SA binding data inferred from the glycan array, we measured elution of the viruses from red blood cells (RBC) possessing different proportions of α2,3- and α2,6-linked SAs. RBC from chicken and human (which are abundant in α2,3-linked SAs), and guinea pig and turkey (which are abundant in α2,6-linked SAs) were used in these tests^17^,^28^,^29^.

Virus elution from chicken, human, and guinea pig RBC at 37 °C (Fig. 3) correlated well with the observed binding to SA on the CFG array. Those viruses with the highest binding profiles on the CFG array, rgHK68 and rgHK68 + 4, eluted from these RBC more slowly than did the “low binding” rgHK68 + 2. The latter completely dissociated from chicken RBC, and partially (to 1 hemagglutination units [HAU]) dissociated from human and guinea pig RBC, after 8 hours of incubation. In contrast, the rgHK68 and rgHK68 + 4 viruses stayed attached at 8 to 16 HAU at this time point. Indeed, rgHK68 never completely eluted from these RBC over the 20-hour course of the experiment. None of the viruses eluted from these RBC at 4 °C after 20 hours (data not shown), a temperature at which the level of the IAV neuraminidase (NA) activity is minimal^30^. Our data therefore suggest that the avidity of rgHK68 + 2 virus to SA receptors present on chicken, human and guinea RBC (as determined at 37 °C) is much lower than that of rgHK68 or rgHK68 + 4. In contrast to chicken, human, and guinea pig RBC, none of the rgHK68 viruses eluted from turkey RBC even after 20 hours of incubation at elevated temperature of 37 °C. Importantly, even though rgHK68 + 2 bound to a limited number of sialylated structures with relatively low affinity, it remained attached to the TRBC for the full 20-hour experiment.

Although each of the rgHK68 viruses contained NAs with identical sequences, it remained possible that different amounts of NA could incorporate into each virion (which might affect the catalytic activity of the virus and therefore the elution rate), or that the changes in HA might indirectly alter NA activity. We therefore compared virus NA activities, relative to both NA and NP content, in a standard fluorometric assay at the same pH (7.2) as was used in the elution tests (Fig. 4). NA activity was similar for rgHK68 and rgHK68 + 2. The rgHK68 + 4 showed slightly higher catalytic activity than did rgHK68 and rgHK68 + 2; however, this effect would increase elution rates while rgHK68 + 4 eluted from chicken, human, and guinea pig RBC much more slowly than did rgHK68 + 2 and, similar to rgHK68 and rgHK68 + 2, did not elute from turkey RBC. Therefore this difference would not be responsible for the slow elution of rgHK68 + 4.

Our data from elution tests with chicken, human, and guinea pig RBC therefore strongly support results from glycan array assays showing a selective impact of HA glycosylation on rgHK68 viruses’ binding to SA-containing receptors. The inability of rgHK68 viruses to elute from turkey RBC suggested that the turkey erythrocytes have a distinct pattern of sialylated receptors compared to the other tested RBC, and indicated a dominant role of cell surface glycan composition in the biological outcome of H3N2 virus binding. The lack of rgHK68 + 2 virus elution from turkey RBC suggests that a low level of binding to limited set of glycans is still sufficient for biologically functional binding when the target cell surface possesses a particular set of sialyl glycans.

### Broad-range high-affinity receptor binding is not a critical determinant of the replication of HK68 viruses in tissue culture and associated cytopathology

We compared growth of rgHK68, rgHK68 + 2, or rgHK68 + 4 viruses in epithelial cell cultures of various origins such as NHBE (primary human bronchial), Calu-3 (human bronchial), A549 (human alveolar), MDCK (canine kidney), and Vero (African Green Monkey kidney) at low (0.01) or high (1.0) multiplicities of infection (MOI) at various times post-infection. We also compared virus replication in Calu-3 cells grown in liquid-covered culture (LCC), which does not produce mucin, or in air-interface culture (AIC), which does produce mucin and most closely mimics the conditions of RT, at temperatures reflective of the proximal (32 °C) and distal (37 °C) airways. The Calu-3 cell line has features of differentiated, functional human airway epithelial cells^31^ (including forming polarized layers with apical and basolateral surfaces and secreting mucin), expresses SA receptors preferred by human H3N2 viruses^32^, and is a well-established human respiratory epithelial cell model for IAV infection^32^,^33^.

There were no consistent significant differences associated with receptor binding or glycosylation observed in the growth kinetics between the three viruses in any of the cell types under any conditions (Fig. 5). The ability to bind a wide range of sialylated glycans with high affinity did not enhance the replication of rgHK68 and rgHK68 + 4 compared to rgHK68 + 2 in cell culture, and low binding did not reduce it for rgHK68 + 2.

To evaluate the cytopathic effect (CPE) associated with viruses’ growth, we examined the zona occludens protein-1 (ZO-1; a major component of tight junction) in Calu-3 LLC monolayers infected with rgHK68 viruses by immunofluorescence microscopy (Fig. 6). In uninfected cells, the ZO-1 protein was localized at the cell-cell boundary in a typical chicken wire-like pattern throughout the monolayer, indicating the presence of intact tight junctions (Fig. 6B). In contrast, extensive CPE was observed in Calu-3 cells infected with each of the viruses, as evidenced by disruption of tight junctions, internalization of ZO-1 protein, and irregularity of the cell margins. As with growth kinetics (Fig. 6A), the three rgHK68 viruses induced comparable level of CPE (Fig. 6B).

### Broad-range-high affinity receptor binding is not a critical determinant of the virulence of HK68 viruses in mice

The virulence of these viruses in mice has previously been studied, but only at high infectious dose of 10^6^ plaque forming unit (PFU) per mouse^11^,^12^. At this high virus dose, there was no correlation between H3N2 virulence and receptor binding.

To more fully test biological effects associated with receptor binding, we infected BALB/c mice intranasally with lower non-lethal doses ranging from 10^2.4^ to 10^5.0^ PFU that may more accurately reflect natural infection doses^34^. Virulence at these lower doses was determined by measuring weight loss and viral lung titers.

Adding either two or four glycosylation sites to the HK68 HA incrementally reduced rgHK68 virus replication and virulence in mice (Fig. 7). As with elution from turkey RBC (Fig. 3) and replication in cultured cells (Fig. 5), the number of sialylated glycans bound and the affinity of binding did not correlate with H3N2 virulence in mice (Fig. 7). Thus, when mice were challenged with 10^5.0^ PFU the mean mouse lung titers of rgHK68 + 4 were up to 1,000-fold lower than titers of rgHK68 (which bound similar levels of SA), while rgHK68 + 2 (which bound much lower levels of SA than either rgHK68 or rgHK68 + 4) produced up to 500-fold higher titers than did rgHK68 + 4 (p < 0.05; Fig. 7A). Similarly, the weight loss (Fig. 7B) of mice infected with rgHK68 was significantly higher than that of mice infected with rgHK68 + 4 (p < 0.05), and mice infected with rgHK68 + 2 lost a similar amount of weight as rgHK68 + 4-infected mice. Challenging with a lower virus dose (10^2.4^ PFU) showed similar effects on mice relative to the higher dose (data not shown).

Collectively, the data from our current study in epithelial cell cultures and mice indicate that modulations of rgHK68 virus receptor binding with additional *N*-linked glycosylation do not translate to such biological consequences as virus virulence.

## Discussion

A decline in the severity of illness and of excess mortality attributed to H3N2 viruses since 1968 has paralleled the progressive addition of *N*-linked glycans to the globular head of HA. We have previously shown that increasing HA glycosylation can reduce H3N2 virulence in mice through virus neutralization by respiratory tract collectin SP-D^11^ and through abrogation of the inflammation associated with activation of ER stress pathways^12^. Glycans on HA can also reduce receptor binding^9^,^10^,^22^,^23^,^26^,^35^, which in turn may affect viral virulence. We therefore tested the connection between glycosylation, receptor binding, and virulence.

**Figure 1:**
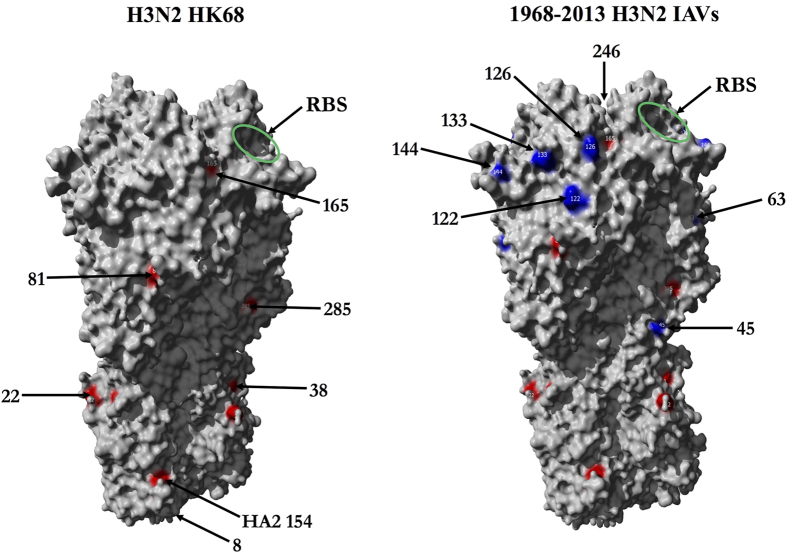
Evolution of H3 *N-*linked glycosylation from 1968–2013. *N-*linked glycosylation sites present on the HA trimer of pandemic H3N2 A/Hong Kong/1/1968 (HK68) (crystal structure PDB 4FNK^7^) are shown in red. Glycosylation sites added to H3 during 1968–2013 are colored in blue. Numbers indicate the start of an *N-*linked glycosylation sequon (Asn) (numbering based on the mature HA molecule after cleavage of the 16-amino acid signal but ignoring the HA1-HA2 cleavage site; residue 483 is HA2 154 in PDB 4FNK). A green oval shows the location of the receptor binding site (RBS).

Increasing HA glycosylation had complex effects on ligand binding by rgHK68 viruses. Adding two glycosylation sites to the HK68 HA (rgHK68 + 2) resulted in reduced binding avidity and the number of recognized sialylated structures, while adding four sites (rgHK68 + 4) enhanced binding compared to rgHK68 + 2. The effect on receptor binding could be due to structural interference between the RBS and carbohydrates. The tri- and tetra-antennary glycans at sites 63 and 126 of rgHK68 + 2 HA^36^ could obstruct the RBS due to their close proximity (within ~10 Å; Fig. 1) and considerable hydrodynamic radii. In the case of rgHK68 + 4, additional glycans at sites 133 and 246, essentially inside the RBS, being primarily substituted with high mannose glycans^36^ could favorably interact sterically in the region which would facilitate restoration of receptor function. Either possibility suggests that the specific glycan position and composition, and not merely the number of glycosylated sites, can influence the recognition of sialylated receptors by H3N2 viruses.

While some studies have found a correlation between receptor binding and IAV virulence^18^,^21^,^22^,^26^, there is growing evidence that the HA binding to SA-containing receptors does not always correlate with infection in itself^37^,^38^,^39^,^40^,^41^,^42^. Consistent with the latter and our previous observation that variations in SA binding by H3N2 IAVs isolated from 1968 to 2012 does not correlate with disease severity or spread^13^, modulation in receptor binding of rgHK68 viruses had no effect on such viral characteristics as elution from turkey RBC, growth in epithelial cell cultures, cytopathology, or virulence in mice. One possible explanation for this may be a role for post-attachment factors^37^,^38^,^39^,^40^,^41^,^42^. Another possibility is that H3N2 binding and entry is mainly dependent on only a limited subset of sialylated glycans, and that binding to these biologically critical ligands has remained relatively constant over time.

This set of critical ligands would be included in the eleven human RT SA recognized by rgHK68 + 2 (Group I of Table 1), a biologically competent virus with weak narrow-specificity binding. Two α2,6-linked SA structures of Group I (Neu5Acα2–6Galβ1–4GlcNAcβ1–3Galβ1–4GlcNAcβ-Sp0 and Neu5Acα2–6Galβ1–4GlcNAcβ1–3Galβ1–4GlcNAcβ1–3Galβ1–4GlcNAcβ-Sp0; glycans 271 and 332, respectively) were recognized by all nineteen H3N2 IAV recombinant HAs (the components of the seasonal influenza vaccines between 1968 and 2012)^14^ as well as by rgHK68 and rgHK68 + 4 (Table 1 and Fig. 8). Moreover, 43 of 45 H3N2 IAVs isolated between 1968 and 2012 recognized these structures^13^ (Fig. 8). Interestingly, most viruses isolated from 1995 till 2012 bound only compounds 271 and 332. The only two isolates that did not recognize these structures (A/BCM/1/1972 and A/OK/5098/1996) also failed to recognize any of the other human RT-associated structures on the CFG array. Although these viruses were both natural isolates from human infection, their virulence and modes of infection have not been further investigated.

Compounds 271 and 332 contain relatively short poly LacNAc and represent poly LacNAc antennae, with the former containing 3.0 repeats and the latter containing 2.5. The poly LacNAc containing structures are widely distributed through all parts of human RT^27^ (Table 1), consistent with a role in H3 IAV pathogenesis. We suggest that glycans 271 and 332 support physiologically relevant binding of H3N2 HA to the human respiratory tract. Binding to other glycans may provide redundancy, without being strictly required for infectivity. Because H3N2 IAVs isolated from 1968 to 2012 bind these relevant structures, we suggest that physiologically relevant receptor binding by H3N2 HA has not changed over the 40 years during which these IAV have circulated in humans. This may help explain the poor correlation between viruses’ receptor binding and pathogenicity, also it implies that receptor binding was not a factor that has contributed to the reduced pathogenicity of recent human seasonal H3N2 viruses.

In addition, the same structures (either as sialosides or as the linear terminal fragments) were the most efficiently recognized structures by all tested recombinant HAs from human IAVs of H1 and H2 subtypes^43^. Thus, our hypothesis of “unmodified physiologically relevant receptor binding” may explain the disconnection between the receptor binding and virulence of IAV of other subtypes as well.

## Methods

### Cells

Epithelial cell lines Madin-Darby canine kidney (MDCK), human alveolar lung adenocarcinoma (A549), African Green Monkey kidney (Vero), and human bronchial lung adenocarcinoma (Calu-3) were purchased from American Tissue Culture Collection (ATCC, Manassas, VA). Primary human bronchial epithelial (NHBE) were purchased from Lonza (Allendale, NJ). MDCK, A549, Vero, and Calu-3 cells were grown in 1× minimum essential medium (MEM) that contained 2 mM L-glutamine, 1 mM sodium pyruvate, 0.1 mM nonessential amino acids, 1.5 g/liter sodium bicarbonate, and 5–10% fetal bovine serum (FBS). NHBE cells were grown in serum-free and hormone-supplemented bronchial epithelial growth medium according to the manufacturer’s procedure. Calu-3 and NHBE cells were seeded onto 24- and 6.5-mm-diameter semipermeable membrane inserts (Corning, NY), respectively, with a final cell density of 3–5 × 10^5^ cells per insert. Cells were grown for 1 week in liquid-covered culture (LCC; Calu-3) or 4–6 weeks in air-interface culture (AIC; Calu-3 and NHBE) with the addition of fresh culture medium every 2 to 3 days, as previously described^31^,^32^,^44^.

### Viruses

Construction of rg H3N2 IAV containing six internal gene segments of H1N1 A/Puerto Rico/8/34 and the HA and NA of HK68, and modification of the HA to generate two mutant variants with additional *N-*linked glycosylation sites at positions 63 and 126 (rgHK68 + 2), or at positions 63, 126, 133, and 246 (rgHK68 + 4), has been previously described^11^. Numbering of glycosylation sites is based on the sequence of the mature HK68 HA molecule without the 16 a.a. signal sequence^6^,^7^. These sites were selected as they reflect changes that appeared naturally during H3N2 IAV evolution at periods of significant antigenic drifts in the 1970s (represented by rgHK68 + 2) and 1995 (represented by rgHK68 + 4)^45^,^46^. The rescued viruses were amplified once in eggs and then grown in MDCK cells for stocks. Stocks were fully sequenced to ensure no inadvertent mutations occurred during rescue or passage. The infectivity of stock viruses was determined by plaque assays or 50% tissue culture infectious dose (TCID_50_) assays as described elsewhere^47^. A mass spectrometry-based platform analysis was used to confirm the occupancy of sites by *N-*linked glycosylation^36^. The usage of chimeric viruses allowed us to exclude the contribution of factors other than additional glycosylation to the observations.

### Hemagglutination and elution assays

Hemagglutination assays were performed using 0.5% chicken, human, and turkey, and 0.75% guinea pig RBC as described elswhere^48^. In the elution tests, each rgHK68 virus was diluted to provide 16 HAU after 1 hour at 4 °C, then the plate was shifted to 37 °C, and elution of viruses from RBC was recorded after 4, 8, and 20 hours of incubation.

### Neuraminidase assays

Cell-grown rgHK68 viruses were concentrated and purified through a gradient of 30% to 50% sucrose in PBS, as described previously^48^. The neuraminidase (NA) activities of the concentrated purified viruses were analyzed with a standard fluorometric assay using 2′-(4-methylumbelliferyl)-α-D-*N-*acetylneuraminic acid (MUNANA; Sigma-Aldrich, Inc., St. Louis, MO) as the substrate^49^, with some modifications. Enzyme kinetic assays were conducted in CMS buffer (0.25 mM CaCl_2_, 0.8 mM MgCl_2_ and 0.15 mM NaCl) pH 7.2 on pre-warmed plates, and read on a SpectraMax M2 microplate reader (Molecular Devices Corp., Sunnyvale, CA) using excitation and emission wavelengths 365 and 460 nm, respectively. The enzyme kinetic data were fit to the Michaelis-Menten equation by using nonlinear regression to establish the Michaelis constant and the maximum velocity of substrate conversion. The NA activities of viruses were then calculated per 1 μg of the virion HA or NP. The amounts of the HA, NA and NP proteins in the sample were determined by 7.5% to 10% sodium dodecyl sulfate-polyacrylamide bis-Tris gel (SDS-PAGE; Invitrogen, Carlsbad, CA) and by Western blot analysis with rabbit antisera containing antibodies against recombinant A/Memphis/1/1971 (H3)-Bel/1942 (N1) for HA, A/Tokyo/3/1967 (H2N2) for NA, and PR8 for NP detection, and secondary goat anti-rabbit alkaline phosphatase conjugated antibodies (Sigma-Aldrich Corp., St. Louis, MO). The imaging was accomplished using Epson Perfection 2400 Photo Scanner (Long Beach, CA), and the densities of the HA, NA or NP bands were analyzed with Gel Eval (v1.37, FrogDance Software, UK).

### Labelling of virus HA

The labelling of viruses with Alexa Fluor-488 (Life Technologies, Rockville, MD) was performed as described previously^13^. Briefly, concentrated purified virus was pelleted out of sucrose and resuspended in a solution containing 0.15 M NaCl, 0.25 mM CaCl_2_, and 0.8 mM MgCl_2_ to about 1.0 × 10^5^ HAU per ml. Then 100 μl of virus suspension was mixed with 10 μl of 1.0 M NaHCO_3_ (pH 9.0), and 0.005 μg of Alexa Fluor-488 succinimidyl ester per HAU was added to mixture. After stirring for 1 hour at room temperature (RT) in the dark, the sample was dialyzed in borate buffered saline containing 0.25 mM CaCl_2_ and 0.8 mM MgCl_2_ (pH 7.2) at 4 °C overnight using Slide-A-Lyzer Mini Dialysis Units 7000 MWCO (Pierce Biotechnology, Rockford, IL). The Alexa-labeled virus HA was confirmed by fluorescence of the HA1 band on a 9% SDS-PAGE gel. Virus binding activity was an additionally evaluated by HA tests to assure that receptor recognition was not affected by the labelling procedure.

### Glycan array assays with Alexa-labelled viruses

**Figure 2:**
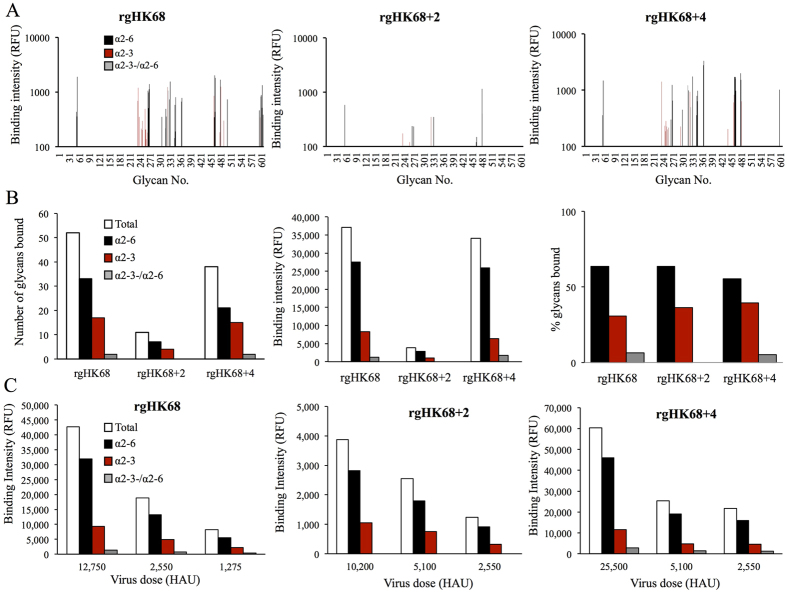
Binding of rgHK68 viruses to sialic acids on a glycan array. (**A**) Binding of rgHK68, rgHK68 + 2, and rgHK68 + 4 viruses to the 61 sialylated glycans that showed binding (see the Methods section for standard experimental conditions). The average from four replicates for each glycan is shown. The glycan numbers (indicated on the horizontal axis) refer to those on version 5.1 of the Consortium for Functional Glycomics printed array. Details of glycan structures can be found at www.functionalglycomics.org; structures for selected glycans are shown in Table 1. (**B**) Total numbers and binding intensities, and proportion of Neu5AC α2,3- and Neu5Acα2,6-linked selected glycans bound by rgHK68 viruses at standard conditions. (**C**) Total binding intensities of rgHK68 viruses to selected glycans at doses ranging from 2,550 HAU to 25,500 HAU (indicated on the horizontal axis). Binding intensities shown in relative fluorescence units (RFU; indicated on the vertical axis).

**Figure 3:**
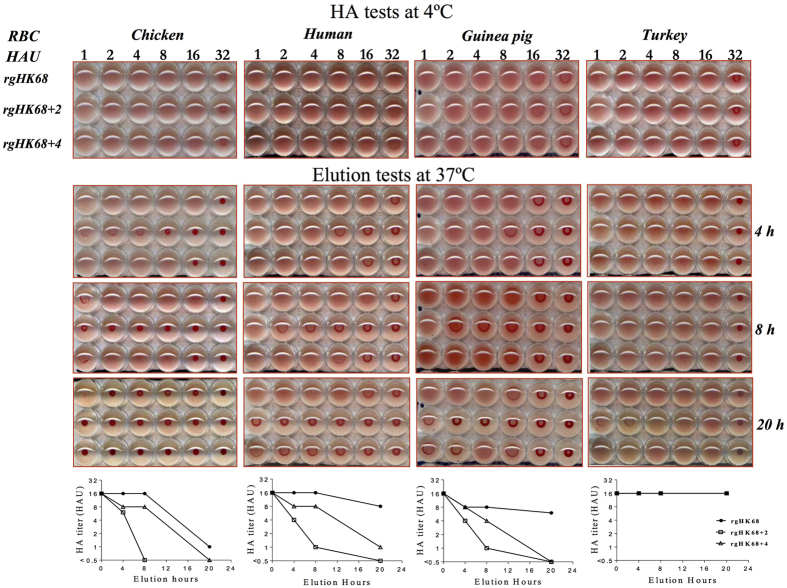
Elution of rgHK68 viruses from red blood cells. In HA assays, the rgHK68, rgHK68 + 2, and rgHK68 + 4 were diluted to provide 16 HAU with 0.5% chicken, or human, or turkey, or 0.75% guinea pig RBC after 1 hour at 4 °C. Then the plates were shifted to 37 °C, and elution of viruses from RBC was recorded after 4, 8, and 20 hours of incubation.

**Figure 4:**
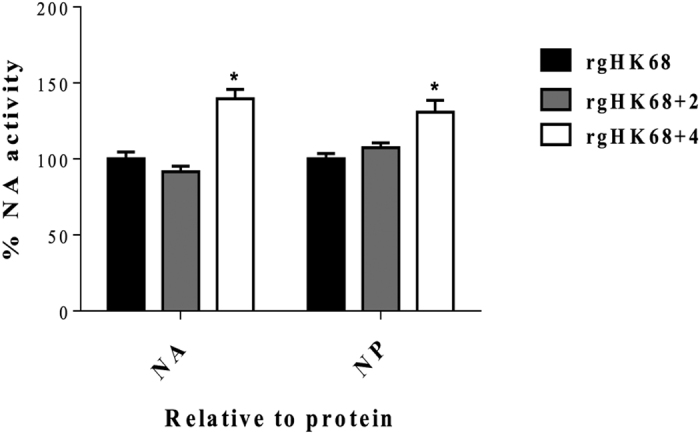
Catalytic activities of rgHK68 viruses. The activity of each viral NA was measured by a standard fluorometric assay and proportioned to the amount of NA or NP in the sample. The NA activities (expressed as NA/NA and NA/NP ratios) of rgHK68 + 2 and rgHK68 + 4 mutants are normalized to those of rgHK68. Error bars indicate the standard deviations (SD) of the mean of the results from three independent experiments. An asterisk indicates a significant difference (p < 0.05) by unpaired Student’s t-test compared with rgHK68 and rgHK68 + 2.

**Figure 5:**
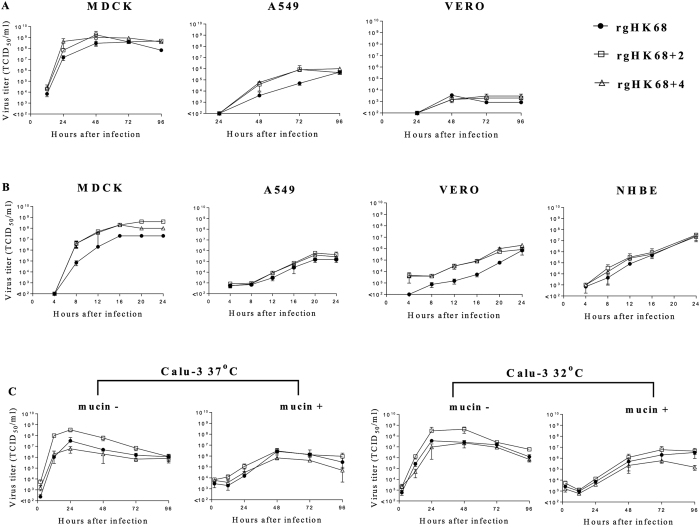
Growth kinetics of rgHK68 viruses in epithelial cell lines. Cell monolayers were infected with rgHK68, or rgHK68 + 2, or rgHK68 + 4 at MOI 0.01 (**A** and **C**) or 1.0 (**B**) at 37 °C (**A**, **B** and **C**) or at 32 °C (**C**). Viruses’ growth in Calu-3 cells (**C**) was examined in the absence or presence of mucin (as indicated). Virus titers were determined for apical culture supernatant fluids at the times indicated on the x-axis. Error bars indicate the SD of the mean of the results from three independent experiments (MDCK, A549, and Vero cell lines) or from three cultures (NHBE and Calu-3 cell lines).

**Figure 6:**
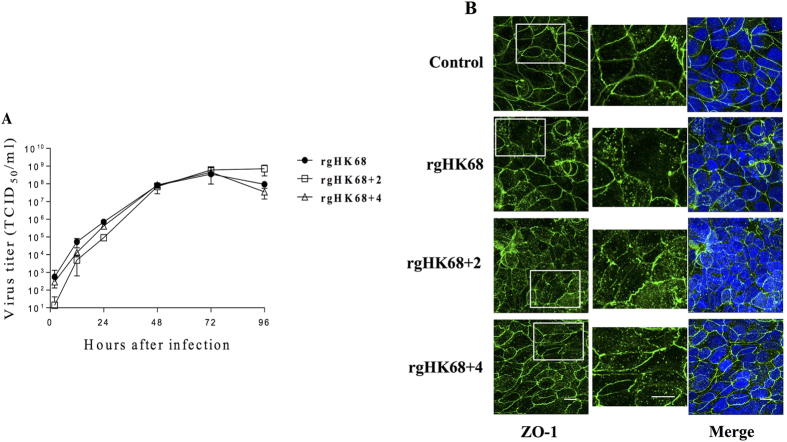
Cell pathology induced by rgHK68 viruses. Monolayers of Calu-3 cells grown in liquid-covered conditions were infected with three viruses at MOI 0.001 and 32 °C. (**A**) Viral growth kinetics. Error bars indicate the SD of the mean of the results from three culture replicates. (**B**) Confocal images of Calu-3 cells stained with antibodies specific for ZO-1 protein (green) 36 hours post-infection. Cell nuclei were visualized with DAPI (blue). Bar, 10 mm. Left column: ZO-1 staining; middle column: higher scale of the ZO-1 region outlined by a box in the left column; right column: ZO-1 staining merged with DAPI. Control: uninfected cell monolayers.

**Figure 7:**
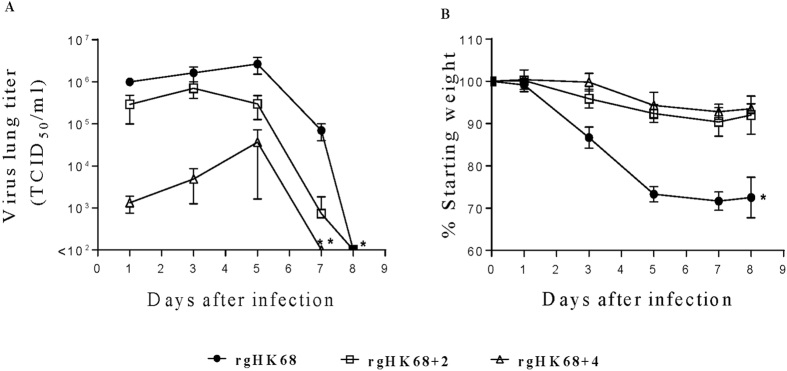
Pathogenicity of rgHK68 viruses in mice. (**A**) Virus lung titers and (**B**) weight loss in BALB/c mice infected with 10^5.0^ PFU per mouse of rgHK68 or rgHK68 + 2 or rgHK68 + 4. (**A**) Growth kinetics of rgHK68 viruses in lungs of mice (n = 3–5 per time point). The mean ± SD are shown. (**B**) Mice (n = 10) were monitored individually for weight loss; results are presented as a mean percentage of starting weight ± SD. (**A,B**) An asterisk indicates a significant difference (p < 0.05) by ANOVA compared with the results for the mice infected with rgHK68 + 2 or rgHK68 + 4. A double asterisk shows a significant difference compared to the results from rgHK68 + 2-infected group.

**Figure 8:**
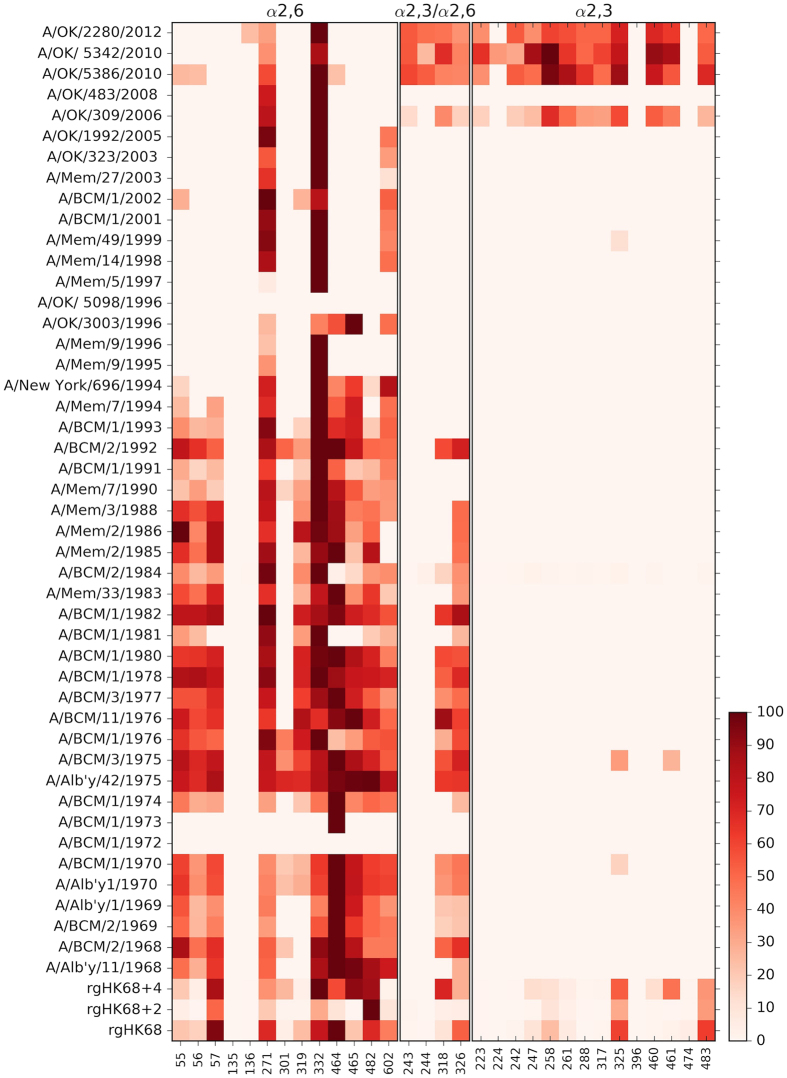
Binding of H3N2 influenza A viruses to receptors of human RT. The heat map of relative binding percent of viruses used in our previous^13^ and current studies to human RT-associated glycans^27^ on version 5.1 of the Consortium for Functional Glycomics printed array is shown, and color-coded from 100 (the highest binding; dark brown) to 0 (the lowest binding; white). Glycans on the horizontal axis are ordered according to α2,6-, α2,3-, or dual α2,3-/α2,6-linkage, and refer to the numbers of the glycans on the printed array. Structures for glycan numbers: 464 - Neu5Acα2-6Galβ1-4GlcNAcβ1-4Mana1-6(GlcNAcβ1-4) (Neu5Acα2-6Galβ1-4GlcNAcβ1-4(Neu5Acα2-6Galβ1-4GlcNAcβ1-2)Mana1-3)Manβ1-4GlcNAcβ1-4GlcNAcβ-Sp21; 465 - Neu5Acα2-6Galβ1-4GlcNAcβ1-6(Neu5Acα2-6Galβ1-4GlcNAcβ1-2)Mana1-6(GlcNAcβ1-4)(Neu5Acα2-6Galβ1-4GlcNAcβ1-2Mana1-3)Manβ1-4GlcNAcβ1-4GlcNAcβ-Sp21; 461 - Neu5Acα2-3Galβ1-4GlcNAcβ1-6(Neu5Acα2-3Galβ1-4GlcNAcβ1-2)Mana1-6(GlcNAβb1-4)(Neu5Acα2-3Galβ1-4GlcNAcβ1-2Mana1-3)Manβ1-4GlcNAcβ1-4GlcNAcβ-Sp21; 460 - Neu5Acα2-3Galβ1-4GlcNAcβ1-4 Mana1-6(GlcNAcβ1-4) (Neu5Acα2-3Galβ1-4GlcNAcβ1-4(Neu5Acα2-3Galβ1-4GlcNAcβ1-2)Mana1-3)Manβ1-4GlcNAcβ1-4GlcNAcβ-Sp21; 474 - Neu5Acα2-3Galβ1-3GlcNAcβ1-6(Neu5Acα2-3Galβ1-4GlcNAcβ1-2)Mana1-6(Neu5Acα2-3Galβ1-3GlcNAcβ1-2Mana1-3)Manβ1-4GlcNAcβ1-4GlcNAcβ-Sp19; 482 - Neu5Acα2-6Galβ1-4GlcNAcβ1-2Mana1-6(Neu5Acα2-6Galβ1-4GlcNAcβ1-2Mana1-3)Manβ1-4GlcNAcβ1-4(Fuca1-6)GlcNAcβ-Sp24; 483 - Neu5Acα2-3Galβ1-4GlcNAcβ1-2Mana1-6(Neu5Acα2-3Galβ1-4GlcNAcβ1-2Mana1-3)Manβ1-4GlcNAcβ1-4(Fuca1-6)GlcNAcβ-Sp24; 57 - Neu5Acα2-6Galβ1-4GlcNAcβ1-2Mana1-6(Neu5Acα2-6Galβ1-4GlcNAcβ1-2Mana1-3)Manβ1-4GlcNAcβ1-4GlcNAcβ-Sp24; 55 - Neu5Acα2-6Galβ1-4GlcNAcβ1-2Mana1-6(Neu5Acα2-6Galβ1-4GlcNAcβ1-2Mana1-3)Manβ1-4GlcNAcβ1-4GlcNAcβ-Sp12; 56 - Neu5Acα2-6Galβ1-4GlcNAcβ1-2Mana1-6(Neu5Acα2-6Galβ1-4GlcNAcβ1-2Man-a1-3)Manβ1-4GlcNAcβ1-4GlcNAcβ-Sp21; 326 - Neu5Acα2-3Galβ1-4GlcNAcβ1-2Mana1-6(Neu5Acα2-6Galβ1-4GlcNAcβ1-2Mana1-3)Manβ1-4GlcNAcβ1-4GlcNAcβ-Sp12; 318 - Neu5Acα2-6Galβ1-4GlcNAcβ1-2Mana1-6(Neu5Acα2-3Galβ1-4GlcNAcβ1-2Mana1-3)Manβ1-4GlcNAcβ1-4GlcNAcβ-Sp12; 325 - Neu5Acα2-3Galβ1-4GlcNAcβ1-2Mana1-6(Neu5Acα2-3Galβ1-4GlcNAcβ1-2Mana1-3)Manβ1-4GlcNAcβ1-4GlcNAcβ-Sp12; 396 - Neu5Acα2-3Galβ1-3GlcNAcβ1-2Mana1-6(Neu5Acα2-3Galβ1-3GlcNAcβ1-2Mana1-3)Manβ1-4GlcNAcβ1-4GlcNAc-Sp19; 319 - Galβ1-4GlcNAcβ1-2Mana1-6(Neu5Acα2-6Galβ1-4GlcNAcβ1-2Mana1-3)Manβ1-4GlcNAcβ1-4GlcNAcβ-Sp12; 301 - Neu5Acα2-6Galβ1-4GlcNAcβ1-2Mana1-6(Galβ1-4GlcNAcβ1-2Mana1-3)Manβ1-4GlcNAcβ1-4GlcNAcβ-Sp12; 332 - Neu5Acα2-6Galβ1-4GlcNAcβ1-3Galβ1-4GlcNAcβ1-3Galβ1-GlcNAcβ-Sp0; 258 - Neu5Acα2-3Galβ1-4GlcNAcβ1-3Galβ1-4GlcNAcβ1-3Galβ1-4GlcNAcb-Sp0; 317 - Neu5Acα2-3Galβ1-4GlcNAcβ1-6(Neu5Acα2-3Galβ1-3)GalNAcα-Sp14; 271 - Neu5Acα2-6Galβ1-4GlcNAcβ1-3Galβ1-4GlcNAcβ-Sp0; 602 - Neu5Acα2-6Galβ1-4GlcNAcβ1-6(Galβ1-3)GalNAcα-Sp14; 288 - Neu5Acα2-3Galβ1-4GlcNAcβ1-6(Galβ1-3)GalNAcα-Sp14; 247 - Neu5Acα2-3Galβ1-3GlcNAcβ1-3Galβ1-4GlcNAcβ-Sp0; 261 - Neu5Acα2-3Galβ1-4GlcNAcβ1-3Galβ1-4GlcNAcβ-Sp0; 243 - Neu5Acα2-6(Neu5Acα2-3Galβ1-3)GalNAcα-Sp8; 244 - Neu5Acα2-6(Neu5Acα2-3Galβ1-3)GalNAcα-Sp14; 242 - Neu5Acα2-3Galβ1-3(6S)GalNAcα-Sp8; 135 - Neu5Acα2-6(Galβ1-3)GalNAcα-Sp8; 136 - Neu5Acα2-6(Galβ1-3)GalNAcα-Sp14; 223 - Neu5Acα2-3Galβ1-3GalNAcα-Sp8; 224 - Neu5Acα2-3Galβ1-3GalNAcα-Sp14.

Glycan binding analysis (in six replicates) was performed by the Protein-Glycan Interaction Core of the CFG, as described^50^. Briefly, 50 μl of various dilutions (ranging from 2,550 HA to 25,500 HAU) of Alexa-labelled viruses in binding buffer pH 7.0 (which inhibits the NA activity) was applied to the printed surface of a slide (v5.1; containing 610 glycans, www.functionalglycomics.org) for 1 hour at 4 °C. The binding image was read in a Perkin-Elmer Microarray XL4000 scanner and analyzed using Imagine image analysis software (v6.0). For comparison, viruses’ binding was assessed at three concentrations (Fig. 2C) and 6 repetitions for each concentration. The binding was then proportioned to a standard 10,200 HAU and averaged. Binding below 100 RFU was considered to be irrelevant.

### Cell culture infection

In growth kinetics assays, cells were infected with rgHK68 viruses at MOI 0.01 or 1.0 in MEM supplemented with bovine serum albumin (BSA) and incubated at 32 °C or 37 °C for the duration of the experiment. Prior to infection, the monolayers of Calu-3 (LCC and AIC) and NHBE (AIC) cells were washed with PBS. In case of NHBE cells, apical secretions were transiently removed. After being washed, cells were supplied with fresh basolateral MEM containing BSA. Virus diluted in PBS was added to the apical surface of cells in a volume of 300 μl for 1.5 hours at 32 °C or 37 °C. Following incubation, monolayers were washed with PBS to remove nonadherent virus and incubated at AIC at designated temperatures for the duration of experiment. At indicated times, apical surfaces of Calu-3 or NHBE cells were washed with 300 μl of MEM-BSA for 30 min at 32 °C or 37 °C.

MDCK, A549, and Vero cells in 24-well plates were infected with rgHK68 viruses for 1 hour at RT. After virus adsorption, cells were washed 3 times with PBS, pH 7.2, then incubated in infection media containing 1 μg/ml acetylated trypsin.

In all cases, tissue culture supernatants were collected at time points ranging from 4 to 96 hours. The amount of virus in the apical washes from Calu-3 and NHBE cells or tissue culture supernatants from MDCK, A549, and Vero cells was determined by TCID_50_ assays in MDCK cells.

### Confocal laser scanning microscopy and immunofluorescence assays

To examine cytopathic effect associated with rgHK68 viruses’ growth, Calu-3 cells (LLC) were infected with each virus at MOI 0.001 for 36 hours. Following infection, monolayers on membrane inserts were washed three times with PBS (pH 7.4) supplemented with Ca^2+^/Mg^2+^, and fixed with 4% formaldehyde for 15 minutes at RT. After being washed, cells were permeabilized in 0.5% Triton X-100 and 0.05% Tween-20, then blocked with 2% PBS-diluted normal goat serum (blocking solution) for 30 min and incubated with primary antibody directed against ZO-1 protein (Cell Signaling Technology, Inc., Danvers, MA) at a 1:250 dilution in blocking solution overnight at 4 °C. After being washed, cells were incubated with secondary Alexa Fluor 488–conjugated antibody (Life Technologies, Rockville, MD) at a 1:1000 dilution in blocking solution for 1 hour at RT in the dark. After a final wash with PBS, cells were mounted with ProLong Gold antifade reagent (Invitrogen, Eugene, OR) containing 4′,6-diamino-2-phenylindole (DAPI) for nuclear staining. Tissue culture filters housing the epithelial cell monolayers were carefully detached from their support and mounted on coverslips. Fluorescence was visualized with an inverted confocal laser-scanning microscope (LSM 710, Zeiss, Germany). Using an automated XY stage control within the Zen software, sections were systematically imaged with a x100 objective lens. One micrometer Z-stacks were captured and maximum intensity volume projections were used for subsequent analysis. Images were further processed using Photoshop CS2 (Adobe, Inc., San Jose, CA).

### Animal studies

Experiments using 8-week-old female BALB/c mice (Jackson Laboratories, Bar Harbor, ME) were performed in a Biosafety Level 2 facility in the Animal Resources Center at St. Jude Children’s Research Hospital (St. Jude; Memphis, TN) and Centers for Disease Control & Prevention (CDC; Atlanta, GA). Animals were given general anesthesia that consisted of 2.5% inhaled isoflurane (Baxter Healthcare Corporation, Deerfield, IL) prior to all interventions. All studies were approved by the respective institutional animal care and use committees at St. Jude and CDC. All methods were performed in accordance with the relevant guidelines and regulations.

To compare the pathogenicity of rgHK68 viruses, mice were infected intranasally (i.n.) with doses of 10^2.4^ and 10^5^ PFU per mouse in 50 μl of sterile PBS. Control mice were administered PBS only. After 1, 3, 5, 7, 8, or 9 days, lungs from 3 to 5 mice from each group were removed under sterile conditions, washed 3 times with PBS, homogenized, and suspended in PBS (total volume, 1 ml). The suspensions for virus titration were centrifuged at 2,000 × *g* for 10 minutes to clear cellular debris. Virus titers were determined by TCID_50_ assays in MDCK cells. Weight changes (calculated for each mouse as a percentage of its weight on day 0 before virus infection) were monitored daily for each group (n = 10) for 21 days after infection.

### Glycan array data processing for human H3N2 isolates

Relative binding of each virus isolate used in our previous study^13^ to each human RT-associated glycan^27^ on the CFG printed array v5.1 was calculated by normalizing the highest RFU value in the subset to 100 for each virus and expressing binding to other glycans as a percentage of that value; in this way the RFUs for three concentrations of virus could be averaged. The heat map of relative binding percent was created using open-source software Python v3.4 and Matplotlib v1.4.

### Statistical analyses

Analysis of variance (ANOVA) followed by Tukey’s multiple comparison test was used to estimate and compare the NA activities, viral titers in the mouse lungs and cell culture supernatants, and weight loss. A p-value < 0.05 was considered significant for these comparisons. Statistical analyses were performed using GraphPad Prism Software (v4.0, GraphPad Software Inc., San Diego, CA).

## Additional Information

**How to cite this article**: Alymova, I. V. *et al*. Glycosylation changes in the globular head of H3N2 influenza hemagglutinin modulate receptor binding without affecting virus virulence. *Sci. Rep.* **6**, 36216; doi: 10.1038/srep36216 (2016).

**Publisher’s note**: Springer Nature remains neutral with regard to jurisdictional claims in published maps and institutional affiliations.

## Figures and Tables

**Table 1:** Binding of rgHK68 viruses in a glycan array^a^.

Group	Glycan N0.	Sialic acid structure	Sialic acid distribution in human RT			Intensity of binding (RFU^b^ ± SD^c^)		
			Nasopharynx and tonsil	Bronchus	Lung	rgHK68	rgHK68 + 2	rgHK68 + 4
I	**57**	**Neu5Acα2-6Galβ1-4GlcNAcβ1-2Mana1-6(Neu5Acα2-6Galβ1-4GlcNAcβ1-2Mana1-3)Manβ1-4GlcNAcβ1-4GlcNAcβ-Sp24**	+		+	**1900** ± **191**	**585** ± **33**	**1492** ± **49**
	237	Neu5Acα2-3GalNAcβ1-GlcNAcβ- Sp0				1190 ± 138	175 ± 44	1420 ± 137
	**258**	**Neu5Acα2-3Galβ1-4GlcNAcβ1-3Galβ1-4GlcNAcβ1-3Galβ1-4GlcNAcb-Sp0**	+	+	+	**490** ± **56**	**120** ± **10**	**215** ± **34**
	266	Neu5Acα2-6GalNAcβ1-GlcNAcβ- Sp0				1062 ± 342	237 ± 101	301 ± 54
	**271**	**Neu5Acα2-6Galβ1-4GlcNAcβ1-3Galβ1-4GlcNAcβ-Sp0**	+	+	+	**1393** ± **71**	**235** ± **30**	**653** ± **184**
	**325**	**Neu5Acα2-3Galβ1-4GlcNAcβ1-2Mana1-6(Neu5Acα2-3Galβ1-4GlcNAcβ1-2Mana1-3)Manβ1-4GlcNAcβ1-4GlcNAcβ-Sp12**	+	+	+	**1230** ± **125**	**351** ± **8**	**930** ± **109**
	**332**	**Neu5Acα2-6Galβ1-4GlcNAcβ1-3Galβ1-4GlcNAcβ1-3Galβ1-4GlcNAcβ-Sp0**	+	+	+	**1550** ± **128**	**350** ± **16**	**1740** ± **156**
	**464**	**Neu5Acα2-6Galβ1-4GlcNAcβ1-4Mana1-6(GlcNAcβ1-4) (Neu5Acα2-6Galβ1-4GlcNAcβ1-4(Neu5Acα2-6Galβ1-4GlcNAcβ1-2)Mana1-3)Manβ1-4GlcNAcβ1-4GlcNAcβ-Sp21**	+			**2008** ± **78**	**121** ± **15**	**1020** ± **208**
	466	Neu5Acα2-6Galβ1-4GlcNAcβ1-6(Neu5Acα2-6Galβ1-4GlcNAcβ1-2)Mana1-6(GlcNAcβ1-4) (Neu5Acα2-6Galβ1-4GlcNAcβ1-4(Neu5Acα2-6Galβ1-4GlcNAcβ1-2)Mana1-3)Manβ1-4GlcNAcβ1-4GlcNAcβ-Sp21				1817 ± 69	150 ± 31	975 ± 349
	**482**	**Neu5Acα2-6Galβ1-4GlcNAcβ1-2Mana1-6(Neu5Acα2-6Galβ1-4GlcNAcβ1-2Mana1-3)Manβ1-4GlcNAcβ1-4(Fuca1-6)GlcNAcβ-Sp24**	+	+	+	**1390** ± **97**	**1147** ± **75**	**1530** ± **40**
	**483**	**Neu5Acα2-3Galβ1-4GlcNAcβ1-2Mana1-6(Neu5Acα2-3Galβ1-4GlcNAcβ1-2Mana1-3)Manβ1-4GlcNAcβ1-4(Fuca1-6)GlcNAcβ-Sp24**	+	+	+	**1250** ± **46**	**403** ± **32**	**635** ± **82**
II	**55**	**Neu5Acα2-6Galβ1-4GlcNAcβ1-2Mana1-6(Neu5Acα2-6Galβ1-4GlcNAcβ1-2Mana1-3)Manβ1-4GlcNAcβ1-4GlcNAcβ-Sp12**	+		+	**433** ± **291**	**50** ± **14**	**358** ± **64**
	236	Neu5Acα2-3GalNAcα-Sp8				688 ± 56	39 ± 18	129 ± 67
	241	Neu5Acα2-3Galβ1-4(Neu5Acα2-3Galβ1-3)GlcNAcβ-Sp8				350 ± 43	13 ± 11	121 ± 46
	**247**	**Neu5Acα2-3Galβ1-3GlcNAcβ1-3Galβ1-4GlcNAcβ-Sp0**	+	+	+	**215** ± **35**	**13** ± **13**	**233** ± **37**
	249	Neu5Acα2-3Galβ1-3GlcNAcβ-Sp0				202 ± 25	10 ± 12	187 ± 22
	251	Neu5Acα2-3Galβ1-4(6S)GlcNAcβ-Sp8				115 ± 43	11 ± 7	283 ± 23
	253	Neu5Acα2-3Galβ1-4(Fuca1-3)GlcNAcβ1-3Galβ1-4(Fuca1-3)GlcNAcβ1-3Galβ1-4(Fuca1-3)GlcNAcβ-Sp0				101 ± 16	2 ± 8	227 ± 33
	270	Neu5Acα2-6Galβ1-4GlcNAcβ1-3Galb1-4(Fuca1-3)GlcNAcβ1-3Galβ1-4(Fuca1-3)GlcNAcβ-Sp0				120 ± 82	99 ± 12	1240 ± 159
	**318**	**Neu5Acα2-6Galβ1-4GlcNAcβ1-2Mana1-6(Neu5Acα2-3Galβ1-4GlcNAcβ1-2Mana1-3)Manβ1-4GlcNAcβ1-4GlcNAcβ-Sp12**	+		+	**218** ± **110**	**45** ± **12**	**1224** ± **99**
	320	GlcNAcβ1-2Mana1-6(Neu5Acα2-6Galβ1-4GlcNAcβ1-2Mana1-3)Manβ1-4GlcNAcβ1-4GlcNAcβ-Sp12				358 ± 31	11 ± 10	992 ± 72
	**326**	**Neu5Acα2-3Galβ1-4GlcNAcβ1-2Mana1-6(Neu5Acα2-6Galβ1-4GlcNAcβ1-2Mana1-3)Manβ1-4GlcNAcβ1-4GlcNAcβ-Sp12**	+		+	**1060** ± **64**	**61** ± **21**	**500** ± **61**
	345	Neu5Acα2-6Galβ1-4GlcNAcβ1-2Mana1-6(Mana1-3)Manβ1-4GlcNAcβ1-4GlcNAc-Sp12				144 ± 37	7 ± 4	360 ± 34
	346	Mana1-6(Neu5Acα2-6Galβ1-4GlcNAcβ1-2Mana1-3)Manβ1-4GlcNAcβ1-4GlcNAc-Sp12				577 ± 39	22 ± 16	795 ± 71
	347	Neu5Acα2-6Galβ1-4GlcNAcβ1-2Mana1-6Manβ1-4GlcNAcβ1-4GlcNAc-Sp12				190 ± 44	11 ± 3	635 ± 82
	348	Neu5Acα2-6Galβ1-4GlcNAcβ1-2Mana1-3Manβ1-4GlcNAcβ1-4GlcNAc-Sp12				808 ± 86	18 ± 10	975 ± 88
	366	Neu5Acα2-6GlcNAcβ1-4GlcNAc-Sp21				663 ± 148	10 ± 5	2792 ± 246
	367	Neu5Acα2-6GlcNAcβ1-4GlcNAcβ1- 4GlcNAc-Sp21				776 ± 151	9 ± 5	3325 ± 529
	441	Neu5Acα2-3Galβ1-4GlcNAcβ1-3 Galβ-Sp8				115 ± 43	4 ± 4	205 ± 10
	462	Neu5Acα2-3Galβ1-4GlcNAcβ1-6(Neu5Acα2-3Galb1-4GlcNAcβ1-2)Mana1-6(GlcNAcβ1-4)(Neu5Acα2-3Galβ1-4GlcNAcβ1-4(Neu5Acα2-3Galβ1-4GlcNAcβ1-2)Mana1-3)Manβ1-4GlcNAcβ1-4GlcNAcβ-Sp21				853 ± 136	20 ± 12	187 ± 39
	463	Neu5Acα2-6Galβ1-4GlcNAcβ12 Mana1-6(GlcNAcβ1-4)(Neu5 Acα2-6Galβ1-4GlcNAcβ1-2Mana1-3)Manb1-4GlcNAcβ1-4GlcNAcβ-Sp21				345 ± 97	6 ± 7	1692 ± 156
	**465**	**Neu5Acα2-6Galβ1-4GlcNAcβ1-6(Neu5Acα2-6Galβ1-4GlcNAcβ1-2)Mana1-6(GlcNAcβ1-4) (Neu5Acα2-6Galβ1-4GlcNAcβ1-2Mana1-3)Manβ1-4GlcNAcβ1-4GlcNAcβ-Sp21**	+			**432** ± **93**	**5** ± **2**	**1600** ± **89**
	481	Neu5Acα2-6Galβ1-4 GlcNAcβ1-6(Neu5Acα2-6Galβ1-4GlcNAcβ1-3)GalNAcα-Sp14				1667 ± 114	52 ± 31	2008 ± 220
	601	Neu5Acα2-6Galβ1-4GlcNAcβ1-3Galβ1-4GlcNAcβ1-6(Galβ1-3) GalNAcα-Sp14				830 ± 149	49 ± 12	1023 ± 104
III	**56**	**Neu5Acα2-6Galβ1-4GlcNAcβ1-2Mana1-6(Neu5Acα2-6Galβ1-4GlcNAcβ1-2Man-a1-3)Manβ1-4GlcNAcβ1-4GlcNAcβ-Sp21**	+		+	**355** ± **50**	**8** ± **4**	**79** ± **25**
	250	Neu5Acα2-3Galβ1-3GlcNAcβ-Sp8				296 ± 50	6 ± 5	41 ± 12
	259	Neu5Acα2-3Galβ1-4GlcNAcβ-Sp0				205 ± 77	15 ± 13	54 ± 16
	260	Neu5Acα2-3Galβ1-4GlcNAcβ-Sp8				208 ± 47	10 ± 4	89 ± 19
	**261**	**Neu5Acα2-3Galβ1-4GlcNAcβ1-3Galβ1-4GlcNAcβ-Sp0**	+	+	+	**138** ± **27**	**44** ± **32**	**95** ± **24**
	264	Neu5Acα2-3Galβ1-4Glcβ-Sp8				180 ± 40	13 ± 4	31 ± 13
	267	Neu5Acα2-6Galβ1-4(6S)GlcNAcβ-Sp8				510 ± 26	20 ± 13	99 ± 27
	268	Neu5Acα2-6Galβ1-4GlcNAcβ-Sp0				378 ± 271	8 ± 10	47 ± 27
	269	Neu5Acα2-6Galβ1-4GlcNAcβ-Sp8				983 ± 123	31 ± 9	99 ± 79
	308	Neu5Acα2-6Galβ1-4GlcNAcβ1-2Mana1-6(GlcNAcβ1-2Mana1-3)Manb1-4GlcNAcβ1-4GlcNAcβ-Sp12				350 ± 43	9 ± 5	60 ± 49
	**319**	**Galβ1-4GlcNAcβ1-2Mana1-6(Neu5Acα2-6Galβ1-4GlcNAcβ1-2Mana1-3)Manβ1-4GlcNAcβ1-4GlcNAcβ-Sp12**	+	+	+	**490** ± **75**	**14** ± **7**	**62** ± **31**
	330	Neu5Acα2-6Galβ1-4GlcNAcβ1-3 Galβ1-3GlcNAcβ-Sp0				727 ± 146	1 ± 3	77 ± 41
	479	Neu5Acα2-3Galβ1-4GlcNAcβ1-6 GalNAcα-Sp14				185 ± 37	8 ± 4	27 ± 16
	491	Neu5Acα2-3Galβ1-3GlcNAcβ1-6 GalNAcα-Sp14				300 ± 50	99 ± 11	18 ± 21
	502	Neu5Acα2-6GalNAcβ1-4(6S) GlcNAcβ-Sp8				730 ± 91	14 ± 7	79 ± 42
	597	Neu5Acα2-6Galβ1-4GlcNAcβ1-3Galβ1-4GlcNAcβ1-3GalNAcα-Sp14				459 ± 84	32 ± 20	41 ± 19
	600	Neu5Acα2-3Galβ1-4GlcNAcβ1-3Galβ1-4GlcNAcβ1-6(Galβ1-3) GalNAcα-Sp14				343 ± 58	45 ± 40	90 ± 37
	**602**	**Neu5Acα2-6Galβ1-4GlcNAcβ1-6 (Galβ1-3)GalNAcα-Sp14**				**880** ± **14**	**111** ± **75**	**39** ± **20**
	605	Neu5Acα2-6Galβ1-4GlcNAcβ1-3Galβ1-4GlcNAcβ1-6(Neu5Acα2-6Galβ1-4GlcNAcβ1-3Galβ1-4 GlcNAcβ1-3)GalNAcα-Sp14				512 ± 90	18 ± 11	13 ± 11
	606	Neu5Acα2-6Galβ1-4GlcNAcβ1-3Galβ1-4GlcNAcβ1-3Galβ1-4GlcNAcβ1-2Mana1-6(Neu5Acα2-6Galβ1-4GlcNAcβ1-3Galβ1-4 GlcNAcβ1-3Gaβb1-4GlcNAcβ1-2Mana1-3)Manβ1-4GlcNAcβ1-4 GlcNAcβ-Sp12				1325 ± 368	12 ± 21	99 ± 104
	608	Neu5Acα2-6Galβ1-4GlcNAcβ1-3Galβ1-4GlcNAcβ1-2Mana1-6 (Neu5Acα2-6Galβ1-4GlcNAcβ1-3 Galβ1-4GlcNAcβ1-2Mana1-3) Manβ1-4GlcNAcβ1-4GlcNAcβ-Sp12				385 ± 152	1 ± 4	70 ± 52
IV	255	Neu5Acα2-3Galβ1-4(Fuca1-3) GlcNAcβ-Sp8				33 ± 39	10 ± 5	200 ± 6
	295	Neu5Acα2-3Galβ1-4GlcNAcβ1-3Galβ1-3GlcNAcβ-Sp0				36 ± 38	5 ± 5	227 ± 19
	**301**	**Neu5Acα2-6Galβ1-4GlcNAcβ1-2 Mana1-6(Galβ1-4GlcNAcβ1-2 Mana1-3)Manβ1-4GlcNAcβ1-4 GlcNAcβ-Sp12**	+	+	+	**93** ± **48**	**16** ± **10**	**450** ± **55**
	459	Neu5Acα2-3Galβ1-4GlcNAcβ1-2 Mana1-6(GlcNAcβ1-4) (Neu5Acα2-3Galβ1-4GlcNAcβ1-2 Mana1-3)Manβ1-4GlcNAcβ1-4 GlcNAcβ-Sp21				16 ± 9	6 ± 2	610 ± 5
	**460**	**Neu5Acα2-3Galβ1-4GlcNAcβ1-4 Mana1-6(GlcNAcβ1-4) (Neu5Acα2-3Galβ1-4GlcNAcβ1-4 (Neu5Acα2-3Galβ1-4GlcNAcβ1-2)Mana1-3)Manβ1-4GlcNAcβ1-4GlcNAcβ-Sp21**	+			**18** ± **20**	**11** ± **8**	**226** ± **70**
	**461**	**Neu5Acα2-3Galβ1-4GlcNAcβ1-6 (Neu5Acα2-3Galβ1-4GlcNAcβ1-2)Mana1-6(GlcNAβb1-4) (Neu5Acα2-3Galβ1-4GlcNAcβ1-2 Mana1-3)Manβ1-4GlcNAcβ1-4 GlcNAcβ-Sp21**	+			**47** ± **41**	**6** ± **6**	**825** ± **23**

^a^Binding of viruses (screened at three concentrations [Fig. 2C] but all normalized to 10, 200 HAU and averaged) was analyzed on the Consortium for Functional Glycomics printed array v5.1 containing 610 glycans (www.functionalglycomics.org). Human RT-associated SA structures are highlighted. Group I glycans were recognized by all three rgHK68 viruses. Group II SAs were recognized by rgHK68 and rgHK68 + 4 only. Groups III and IV SAs were recognized only by rgHK68 or rgHK68 + 4, respectively. Binding below 100 RFU was regarded as irrelevant.

^b^RFU–relative fluorescent units.

^c^SD–standard deviation.
